# Juglanthraquinone C Induces Intracellular ROS Increase and Apoptosis by Activating the Akt/Foxo Signal Pathway in HCC Cells

**DOI:** 10.1155/2016/4941623

**Published:** 2015-11-22

**Authors:** Ya-Qin Hou, Yao Yao, Yong-Li Bao, Zhen-Bo Song, Cheng Yang, Xiu-Li Gao, Wen-Jing Zhang, Lu-Guo Sun, Chun-Lei Yu, Yan-Xin Huang, Guan-Nan Wang, Yu-Xin Li

**Affiliations:** ^1^National Engineering Laboratory for Druggable Gene and Protein Screening, Northeast Normal University, Changchun 130024, China; ^2^Research Center of Agriculture and Medicine Gene Engineering of Ministry of Education, Northeast Normal University, Changchun 130024, China; ^3^Institute of Genetics and Cytology, Northeast Normal University, Changchun 130024, China; ^4^Department of Hematology, The Affiliated Hospital of Xuzhou Medical College, Xuzhou, Jiangsu 221002, China

## Abstract

Juglanthraquinone C (JC), a naturally occurring anthraquinone extracted from *Juglans mandshurica*, could induce apoptosis of cancer cells. This study aims to investigate the detailed cytotoxicity mechanism of JC in HepG2 and BEL-7402 cells. The Affymetrix HG-U133 Plus 2.0 arrays were first used to analyze the mRNA expression exposed to JC or DMSO in HepG2 cells. Consistent with the previous results, the data indicated that JC could induce apoptosis and hyperactivated Akt. The Western blot analysis further revealed that Akt, a well-known survival protein, was strongly activated in HepG2 and BEL-7402 cells. Furthermore, an obvious inhibitory effect on JC-induced apoptosis was observed when the Akt levels were decreased, while the overexpression of constitutively active mutant Akt greatly accelerated JC-induced apoptosis. The subsequent results suggested that JC treatment suppressed nuclear localization and increased phosphorylated levels of Foxo3a, and the overexpression of Foxo3a abrogated JC-induced apoptosis. Most importantly, the inactivation of Foxo3a induced by JC further led to an increase of intracellular ROS levels by suppressing ROS scavenging enzymes, and the antioxidant *N*-acetyl-L-cysteine and catalase successfully decreased JC-induced apoptosis. Collectively, this study demonstrated that JC induced the apoptosis of hepatocellular carcinoma (HCC) cells by activating Akt/Foxo signaling pathway and increasing intracellular ROS levels.

## 1. Introduction

*Juglans mandshurica* Maxim (Juglandaceae) is one of the rare species of trees used as a traditional medicine, and many studies have reported on the screening of apoptosis-inducing compounds isolated from* J. mandshurica* [1, 2]. Juglone, a major chemical constituent of* J*.* mandshurica* Maxim [3], induces the increase of intracellular reactive oxygen species (ROS) levels, mitochondrial dysfunction, and elevated ratio of Bax/Bcl-2, triggering events responsible for mitochondrial-dependent apoptosis in human leukemia cell HL-60 [4, 5]. Plumbagin, another naphthoquinone, reduces a change in Bcl-2/Bax ratios, resulting in mitochondrial membrane potential loss, Cytochrome* c* release, and caspase-9 activation, triggering the mitochondrial apoptosis [6]. Juglanthraquinone C (JC), a new naturally occurring anthraquinone compound isolated from the stem bark of* J. mandshurica*, was reported to have significant anticancer effects by inducing S-phase arrest and mitochondrion-dependent apoptosis [7]. However, the underlying signal transduction pathways that mediated JC-induced cell apoptosis were still unknown.

The induction of apoptosis is a major mechanism of cancer therapeutics, and it is a constitutive suicide program triggered by a variety of extrinsic and intrinsic signals. The tumor necrosis factor (TNF) acts via the tumor necrosis factor receptor (TNFR) and is a part of the extrinsic pathway for triggering apoptosis [8]. TNFR can recruit the adaptor proteins Fas-associated death domain (FADD) that can trigger the caspase cascade, irreversibly sensitizing the cell to apoptosis [9]. Mitochondrial apoptosis is the best-known intrinsic apoptosis pathway [10]. Mitogen-activated protein kinase (MAPK) signaling pathways, including extracellular signal-regulated protein kinase 1/2 (ERK1/2),* c*-Jun N-terminal kinase (JNK), and p38 MAPK (p38), can trigger mitochondrial apoptosis. High glucose also can induce apoptosis in HepG2 cells through activating the ASK1-p38/JNK pathway [11].

Akt or protein kinase B, a 57-kDa Ser/Thr kinase, is activated by extracellular signals. Akt is frequently activated in cancer cells, and its activation promotes cell proliferation and provides protection from apoptosis [12]. But hyperactivated Akt induces premature senescence and sensitizes cells to ROS-mediated apoptosis by increasing intracellular ROS through increased oxygen consumption and by inhibiting the expression of ROS scavengers downstream of Foxo [13]. Foxo is directly phosphorylated by Akt, and then its transcriptional activity is inhibited. Foxo3a is a member of forkhead transcription factors (Foxos) and plays an important role in protecting cells against oxidative stress through regulating ROS scavengers, including superoxide dismutase 2 (SOD2) and catalase. In normal cells, low amounts of ROS are eliminated by nonenzymatic and enzymatic antioxidizing agents such as glutathione, thioredoxin, SOD2, catalase, and peroxidases [14]. So the inhibition of ROS scavenger activation could cause an increase of ROS levels. High levels of ROS cause changes in cellular adenosine triphosphate (ATP) and Ca^2+^ levels and lead to the release of Cytochrome* c* and mitochondrion-dependent apoptosis [15].

Hepatocellular carcinoma (HCC) constitutes one of the most prevalent malignant diseases. The purpose of this study is to clarify the molecular mechanisms by which JC induced the apoptosis of HepG2 and BEL-7402 cells. Interestingly, JC was found to induce mitochondrion-dependent apoptosis by activating the Akt/Foxo signaling pathway, resulting in the apoptosis of HCC cells; this was contradictory to the conventional role of Akt in apoptosis. Further studies revealed that the hyperactive Akt induced by JC inhibited Foxo transcription factors, impaired ROS scavenging, and eventually resulted in the apoptosis of HCC cells.

## 2. Materials and Methods

### 2.1. Chemicals, Antibodies, Kits, and Reagents

JC was isolated from the stem bark of* J. mandshurica*, and its chemical structure was described by Lin et al. [16]. The purity of JC was greater than 98% as determined by the high-performance liquid chromatography-mass spectrometry. Antibodies against p65, p38, p-p38, JNK, p-JNK, and Histone H1 were purchased from Santa Cruz Biotechnology (CA, USA). Antibodies against ERK, p-ERK, Akt, p-Akt (Ser473), caspase-9, cleaved caspase-9, caspase-3, cleaved caspase-3, Foxo3a, p-Foxo3a, and catalase were purchased from Cell Signaling Technology (MA, USA). Antibody against SOD2 was purchased from EMD Millipore Corporation Division (MA, USA). A mouse monoclonal antibody against glyceraldehyde-3-phosphate dehydrogenase (GAPDH) was purchased from KangChen Bio-tech (Shanghai, China). Dulbecco's modified Eagle's medium (DMEM), RPMI medium 1640 (1640),* N*-acetyl-L-cysteine (NAC), PEG-catalase, 3-(4,5-dimethylthiazol-2-yl)-2,5-diphenyl-2H-tetrazolium bromide (MTT), and phosphatidylinositol-3-kinase (PI3 K) inhibitor LY294002 were purchased from Sigma Chemical (MO, USA). Dimethyl sulfide (DMSO) was purchased from Ameresco (MA, USA). The one-step TUNEL apoptosis assay kit, phorbol 12-myristate 13-acetate (PMA), ROS assay kit, and fluorescence probe dihydroethidium (DHE) were purchased from Beyotime Institute of Biotechnology (Shanghai, China). The Annexin V-FITC Apoptosis Detection Kit was purchased from Becton, Dickinson and Company (NJ, USA). An Akt siRNAs Kit, including negative control (Nc), GAPDH positive control, 438, and 1191, was purchased from GenePharma (China).

### 2.2. Cell Culture

Human HCC cell lines HepG2 and BEL-7402 were purchased from the Cell Bank of the Chinese Academy of Sciences (Shanghai, China). HepG2 cells were cultured in DMEM and the BEL-7402 cells in 1640. Both of them were supplemented with 10% fetal bovine serum (FBS) (Sijiqing, China) at 37°C with 5% CO_2_.

### 2.3. Cytotoxicity and Proliferation Assay

Cytotoxic effects of JC on BEL-7402 cells were tested by the MTT cell viability assay. Briefly, BEL-7402 cells were plated in 96-well plates (8 × 10^3^ cells/well) and routinely cultured for 12 hours. Then, the cells were treated with various concentrations of JC or DMSO in a medium supplemented with 3% FBS. After 48 hours of treatment, the cells were analyzed by the MTT assay (as described in [7]).

### 2.4. siRNA Transfection

HepG2 and BEL-7402 cells (2 × 10^5^) were seeded into each well of 12-well plates with an appropriate complete growth medium 24 hours prior to transfection. Cells were incubated at 37°C with 5% CO_2_ overnight and treated with 150 pmol siRNA per well. The siRNAs were incubated with the Lipofectamine 2000 Transfection Reagent, which was purchased from Invitrogen (CA, USA), according to the manufacturer's instructions. After incubation for 12 hours, cells were washed twice with phosphate-buffered saline (PBS), and the medium was replaced by another medium with 3% FBS and JC. Then HCC cells were harvested and assayed. This study included two human siRNAs (GenePharma, China) designed against Akt (GenBank NM_001288.4) and two control siRNAs. The sequences for control siRNAs and Akt siRNAs were as follows: negative control (sense: 5′-UUCUCCGAACGUGUCACGUTT-3′, antisense: 5′-ACGUGACACGUUCGGAGAATT-3); GAPDH positive control (sense: 5′-GUAUGACAACAGCCUCAAGTT-3′, antisense: 5′-CUUGAGGCUGUUGUCAUACTT-3′); Akt siRNA 1191 (sense: 5′-GACGGGCACAUUAAGAUCATT-3′, antisense: 5′-UGAUCUUAAUGUGCCCGUCTT-3′); and Akt siRNA 438 (sense: 5′-GCACCUUCAUUGGCUACAATT-3′, antisense: 5′-UUGUAGCCAAUGAAGGUGCTT-3′).

### 2.5. Plasmid Construction and Transfection

Human full-length Akt and Foxo3a were cloned into the pcDNA3 basic vector. Akt (S473D) and Akt (S473A), which contain mutations at the phosphorylation site (Ser473 to alanine or aspartate), were generated by site-directed mutagenesis. The primer sequences used were as follows: Akt (S473D) (sense: 5′-CCGCTGGCCGAGTAGTCGAACTGGGGGAAGTG-3′, antisense: 5′-CACTTCCCCCAGTTCGACTACTCGGCCAGCGG-3′); Akt (S473A) (sense: 5′-CGCTGGCCGAGTAGGCGAACTGGGGGAAGTG-3′, antisense: 5′-CACTTCCCCCAGTTCGCCTACTCGGCCAGCG-3′). To knockdown the expression of Akt, two Akt short interfering RNA (shRNA) vectors were created. The two shRNA targeting Akt sequences were synthesized by GenScript. The shRNA sequences targeting Akt were as follows: Akt#1 (sense: 5′-GATCCGCTACTTCCTCCTCAAGAATGTTCAAGAGACATTCTTGAGGAGGAAGTAGCTTTTTTGGAAA-3′, antisense: 5′-AGCTTTTCCAAAAAAGCTACTTCCTCCTCAAGAATGTCTCTTGAACATTCTTGAGGAGGAAGTAGCG-3′); Akt#2 (sense: 5′-GATCCGCTGGAGAACCTCATGCTGTTCAAGAGACAGCATGAGGTTCTCCAGCTTTTTTGGAAA-3′, antisense: 5′-AGCTTTTCCAAAAAAGCTGGAGAACCTCATGCTGTCTCTTGAACAGCATGAGGTTCTCCAGC-G-3′). The oligonucleotides were inserted into the pRNAT-U6.1/Hygro vector, which expresses hairpin sequences utilizing the U6 RNA pol III promoter (GenScript Corporation, NJ, USA). All constructs were confirmed by DNA sequencing. HepG2 and BEL-7402 cells (4 × 10^5^ or 2 × 10^5^) per well were seeded in 6-well plates or 12-well plates, respectively. After incubating overnight with a medium containing 10% FBS, the aforementioned different plasmids were transfected into the cells using Lipofectamine 2000, according to the manufacturer's instructions.

### 2.6. Apoptosis Analysis by DAPI Staining

JC-induced apoptosis was detected by DAPI staining. The cells were harvested and stained with DAPI (as described in [17]). Images were acquired using an Olympus IX71 fluorescence microscope (Olympus, Tokyo, Japan). Apoptotic cells were morphologically defined by chromatin condensation and fragmentation.

### 2.7. Apoptosis Analysis by TUNEL Staining

Commercially available one-step TUNEL apoptosis assay kits (BD Biosciences, NJ, USA) were used to evaluate the apoptotic response of HepG2 cells, according to the manufacturer's instructions. HepG2 cells were stained with DAPI (as described in [17]).

### 2.8. Apoptosis Analysis by Annexin V-FITC/PI Double Staining

The apoptosis and necrosis ratio was analyzed using the Annexin V-FITC Apoptosis Detection Kit, according to the manufacturer's instructions. Fluorescence was measured by flow cytometer (BD Biosciences).

### 2.9. Western Blot Analysis

The cells were harvested, and cytosolic and nuclear extracts were prepared. Western blotting was performed as described in [18]. The membranes were incubated overnight at 4°C with primary antibodies specific for p65, p38, p-p38, JNK, p-JNK, ERK, p-ERK, Akt, p-Akt (Ser473), caspase-9, cleaved caspase-9, caspase-3, cleaved caspase-3, Foxo3a, p-Foxo3a, catalase, SOD2, GAPDH, and Histone H1. The membranes were subsequently visualized using an ECL reagent (TransGen Biotech, China) and analyzed with DNR Bio-Imaging Systems (Israel).

### 2.10. ROS Measurement

The production of ROS was monitored using the nonfluorescent probe 2′,7′-dichlorodihydrofluorescein diacetate (DCFH-DA). DCFH-DA diffuses into cells and is deacetylated by esterases to form the nonfluorescent product 2′,7′-dichlorodihydrofluorescein (DCFH). In the presence of ROS, DCFH reacts with ROS to form the fluorescent product 2′,7′-dichlorofluorescein (DCF). HepG2 cells were treated with 8 *μ*g/mL of JC for 12, 24, 36, and 48 hours. BEL-7402 cells were treated with 6.7, 8.7, and 1.05 *μ*g/mL of JC for 12 hours. DCFH-DA was diluted in a medium at a final concentration of 10 *μ*M and incubated with microglia for 30 minutes at 37°C in the dark. The cells were washed twice with PBS, trypsinized, and then resuspended in 250 *μ*L of PBS. Fluorescence was measured at an emission wavelength of 530 nm and an excitation wavelength of 485 nm by flow cytometry.

The intracellular ROS levels of HepG2 cells were assayed using the fluorescence probe DHE. Intracellular DHE is oxidized to ethidium, which binds to DNA and stains nuclei with a bright fluorescent red color. HepG2 cells were seeded in 6-well plates and pretreated with JC as previously described. DHE was diluted in a medium at a final concentration of 10 *μ*M and incubated with microglia for 30 minutes at 37°C in the dark. The cells were washed twice with PBS, trypsinized, and then resuspended in 250 *μ*L of PBS. Fluorescence was measured at an emission wavelength of 370 nm and an excitation wavelength of 420 nm by flow cytometry.

### 2.11. RNA Extraction and Affymetrix Microarray

HepG2 cells were seeded at 3.5 × 10^5^ cells per well in 6-well plates and treated with DMSO or 8 *μ*g/mL of JC for 4 or 10 hours. The total RNA was isolated from HepG2 cells using the RNeasy Mini Kit (Qiagen, Hilden, Germany), following the manufacturer's protocol. The RNA quantity and quality were assessed by e-Spect (Malcom, Tokyo, Japan). The 260/280 ratios of all samples were between 2.13 and 2.18. The total RNA (50 ng) were used to generate amplified and biotinylated cRNA. Affymetrix HG-U133 Plus 2.0 arrays (Affymetrix, CA, USA) were hybridized for 16 hours in a 45°C incubator, rotated at 60 rpm, and washed and stained according to the Affymetrix GeneChip Expression Analysis Manual. Finally, Affymetrix HG-U133 Plus 2.0 arrays were scanned using the GeneChip Scanner 3000 7G (Affymetrix).

### 2.12. Fold Change and Gene Ontology Analysis

To screen out the differentially expressed genes between the treatment group and the control group, the CEL files were analyzed. The raw data was normalized using the robust multiarray average method [19, 20]. Fold changes of gene expression ≥1.5 were considered significant and used in the following Gene Ontology (GO) analysis [21]. GO term scores with *P* ≤ 0.05 were considered significant. The GO analysis was performed using the Database for Annotation, Visualization and Integrated Discovery (DAVID) (http://david.abcc.ncifcrf.gov). Heat maps were made using the freely available statistical computing software R (http://mirror.bjtu.edu.cn/cran/). A probe set is a group of probe pairs used together to interrogate a sequence that represents a gene on the array. The median value of several probe sets, which represent one gene, was taken.

### 2.13. Statistical Analysis

Experiments were repeated at least three times. Statistical analysis of the data was performed using the Student *t*-test and two-tailed distribution. The significance level was set at ^*∗*^ *P* < 0.05 and ^*∗∗*^ *P* < 0.01. Error bars denote the standard deviation.

## 3. Results

### 3.1. Role of JC in Inducing Apoptosis

**Figure 1:**
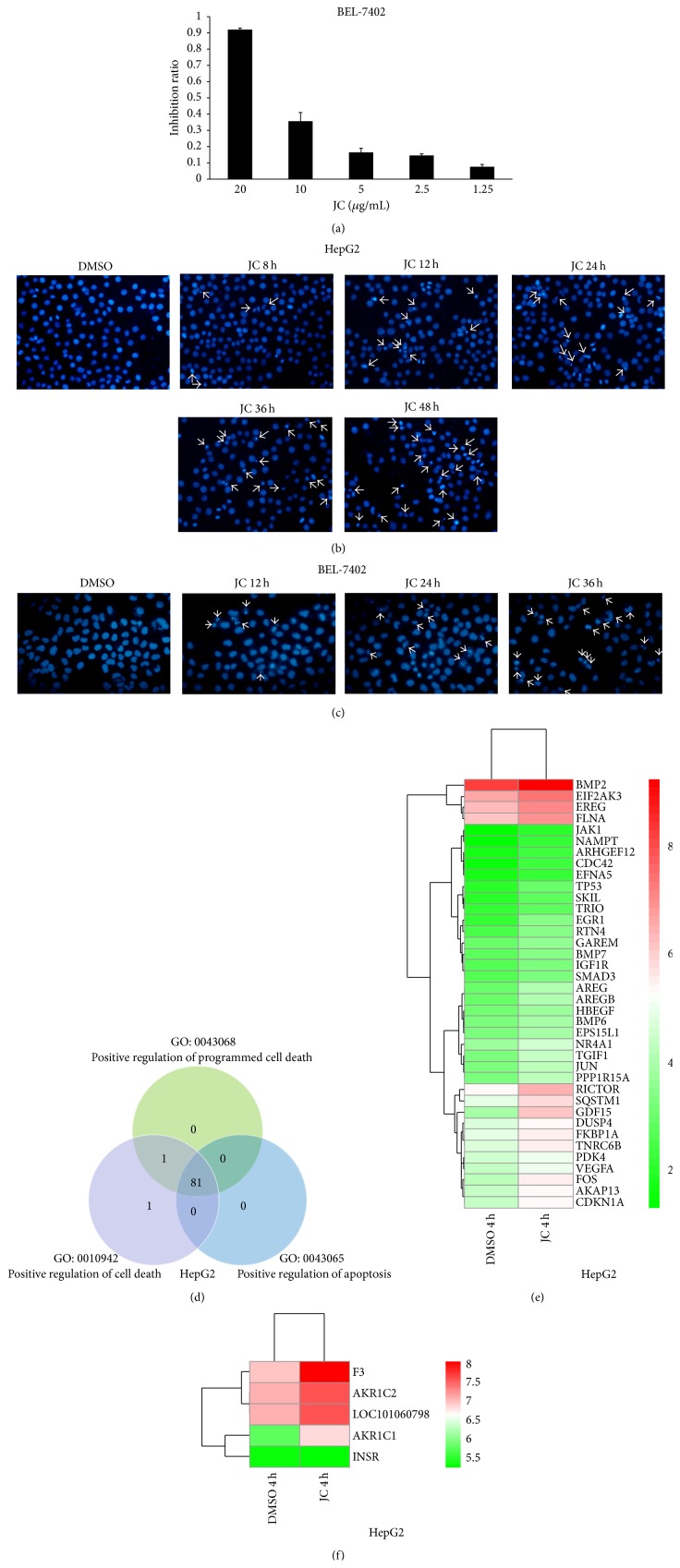
JC induces apoptosis. (a) BEL-7402 HCC cells were treated with indicated concentrations of JC for 48 hours and then subjected to the MTT cell viability assay. (b, c) Detection of apoptosis in JC-induced HCC cells by DAPI staining. HepG2 cells were treated with DMSO or 8 *μ*g/mL of JC for the indicated times (b). BEl-7402 cells were treated with 8.7 *μ*g/mL of JC for the indicated times (c). Then, the cells were fixed and stained with DAPI. Arrows are used to indicate apoptotic bodies in apoptotic HCC cells. (d) DNA microarray analysis of the genes enriched in cell death, programmed death, and apoptosis after HepG2 cells were treated with JC for 10 hours. (e) GO classification for upregulated genes after HepG2 cells were treated with JC for 4 hours. (f) Clustering analysis of genes that can positively regulate Akt signaling after HepG2 cells were treated with JC for 4 hours.

Previous studies have suggested that JC showed strong cytotoxicity in HepG2 cells. In this study, JC was found to reduce the cell viability of HCC BEL-7402 cells in a dose-dependent manner (Figure 1(a)). For a 48-hour exposure, the IC50 was 10.5 *μ*g/mL in BEL-7402 cells. Some previous studies have suggested that JC could selectively inhibit cancer cell viability by inducing apoptosis. To further confirm the ability of JC to induce the apoptosis of human liver cancer cells, the chromatin condensation and DNA fragmentation by DAPI staining in HepG2 and BEL-7402 cells were analyzed (Figures 1(b) and 1(c)). HCC cells exposed to JC showed chromatin condensation and fragmented nuclei in a time-dependent manner.

To study the impact of JC-induced cytotoxicity on gene expression and reveal the mechanisms responsible for JC-induced apoptosis in HepG2 cells, the mRNA expression was analyzed by Affymetrix HG-U133 Plus 2.0 arrays. Genes with an expression ratio ≥1.5-fold were regarded as differentially transcribed genes [21]. After treating HepG2 cells with JC for 10 hours, 2494 individual probe sets were differentially expressed, and 1271 of these 2494 probe sets were downregulated while 1223 were upregulated due to JC treatment.

To identify different gene clusters among the differentially transcribed genes, the DAVID Functional Annotation Tool was used for GO analysis. After treating HepG2 cells with JC for 10 hours, the data of probe sets, which were upregulated by JC, were enriched by GO. Based on this analysis, three main clusters encoding genes involved in cell death, including GO: 0043068, positive regulation of programmed cell death (82 probe sets); GO: 0010942, positive regulation of cell death (83 probe sets); and GO: 0043065, positive regulation of apoptosis (81 probe sets), were identified in the upregulated data of the JC treatment group. Moreover, 81 probe sets were shared by the three main cluster-enriched genes that were upregulated by JC (Figure 1(d)). These data further verified that JC could induce apoptosis of HepG2 cells, and the results were consistent with a previous study [7].

### 3.2. Activation of Akt Signaling Pathway Caused by JC

To investigate the differentially expressed genes involved in signaling pathways that mediated JC-induced apoptosis, the mRNA expression was analyzed after treating HepG2 cells with JC for 4 hours. The results of this study suggested that 1113 individual probe sets were differentially expressed after treating HepG2 cells with JC for 4 hours. Out of these 1113 probe sets, 482 were downregulated and 631 were upregulated due to JC treatment. The probe sets, which were upregulated, were used for GO analysis. A significant GO term (GO: 0007167, enzyme-linked receptor protein signaling pathway) was found. As shown in Figure 1(e), out of the upregulated probe sets, 54 were involved in the enzyme-linked receptor protein signaling pathway. An enzyme-linked receptor, also known as a catalytic receptor, is a transmembrane receptor, and the binding of an extracellular ligand triggers an enzymatic activity on the intracellular side. Receptor tyrosine kinases (RTKs) are the main types of enzyme-linked receptors, and MAPK cascade, nuclear factor kappa-light-chain-enhancer of activated B cells (NF-*κ*B), and PI3 K/Akt are the main signaling pathways triggered by RTKs [22–24]. Surprisingly, four genes related to the PI3 K/Akt signaling pathway were found to be significantly upregulated in HepG2 cells treated with JC compared to those treated with DMSO (Figure 1(f)). Importantly, it was found that all of the four genes and one uncharacterized probe set play a role in positively regulating the Akt signaling pathway.

**Figure 2:**
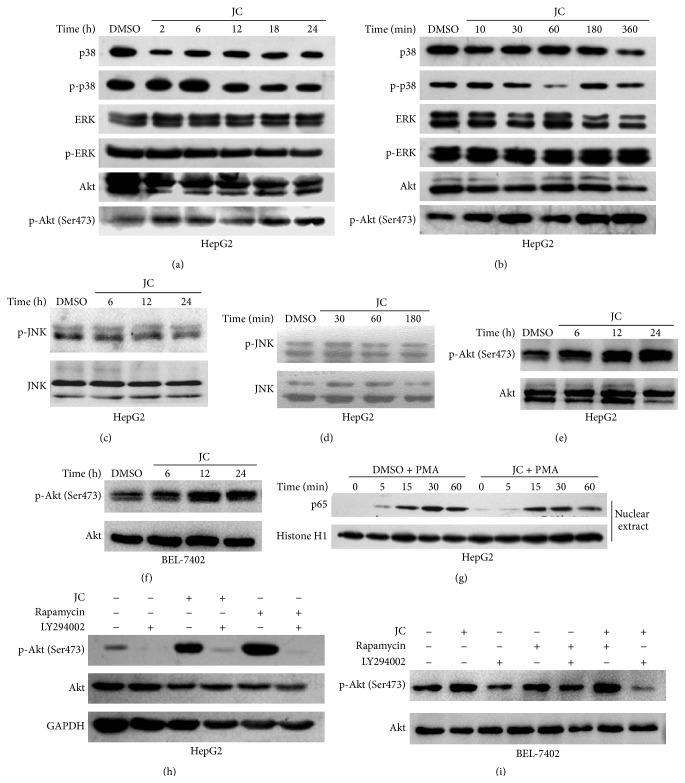
JC activates Akt signaling pathway. (a–d) Effects of JC on MAPK and Akt signaling pathways were determined by Western blot analysis. HepG2 cells were treated with either DMSO or 8 *μ*g/mL of JC for different time periods. (e, f) Effects of JC on the Akt signaling pathway were determined by Western blot analysis. HepG2 cells were cultured in a medium without FBS for 12 hours and then treated with either DMSO or 8 *μ*g/mL of JC for the indicated times (e). BEL-7402 cells were treated with either DMSO or 8.7 *μ*g/mL of JC for the indicated times (f). (g) Effects of JC on the NF-*κ*B signaling pathway were determined by Western blot analysis. HepG2 cells were treated with either DMSO or 8 *μ*g/mL of JC for 12 hours and then treated with PMA, which is an activator of NF-*κ*B, for 5, 15, 30, and 60 minutes. Histone H1 was used as a loading control. (h, i) Effects of LY294002 and JC on Akt activation were determined by Western blot analysis. HepG2 cells were pretreated with 50 *μ*M of LY294002 for 1 hour and then treated with 15 nM of rapamycin and 8 *μ*g/mL of JC separately for 3 hours (h). BEL-7402 cells were treated with 30 *μ*M of LY294002 for 1 hour and then treated with 15 nM of rapamycin and 8.7 *μ*g/mL of JC separately for 3 hours (i). GAPDH was used as a loading control.

To validate the microarray data, the effects of JC on NF-*κ*B, MAPK, and Akt signaling pathways were analyzed by Western blot. Compared to the control, JC had no obvious effect on the nuclear translocation of p65 and the phosphorylated level of p38, JNK, and ERK; however, the level of Akt phosphorylation on Ser473, which has been shown to be an important driver of human cancer [25], was greatly increased (Figures 2(a)–2(d) and 2(g)). After culturing in a medium without FBS for 12 hours, JC induced a higher level of Akt phosphorylation (Figure 2(e)). Also, Akt was found to be activated in BEL-7402 cells after being treated with JC (Figure 2(f)).

To further determine the activation of Akt after JC treatment, HepG2 and BEL-7402 cells were pretreated with the PI3 K inhibitor LY294002 for 1 hour and then treated with JC. In addition, rapamycin, a selective mTOR inhibitor that could induce the activation of Akt signaling through an IGF-1R-dependent mechanism [26], was used as a positive control. The increased level of Akt phosphorylation induced by JC was found to be dramatically reversed by LY294002 (Figures 2(h) and 2(i)). Collectively, these data indicated that the PI3 K/Akt signaling pathway was activated by JC.

### 3.3. Effect of Akt Deficiencies on JC-Induced Apoptosis of HCC Cells

**Figure 3:**
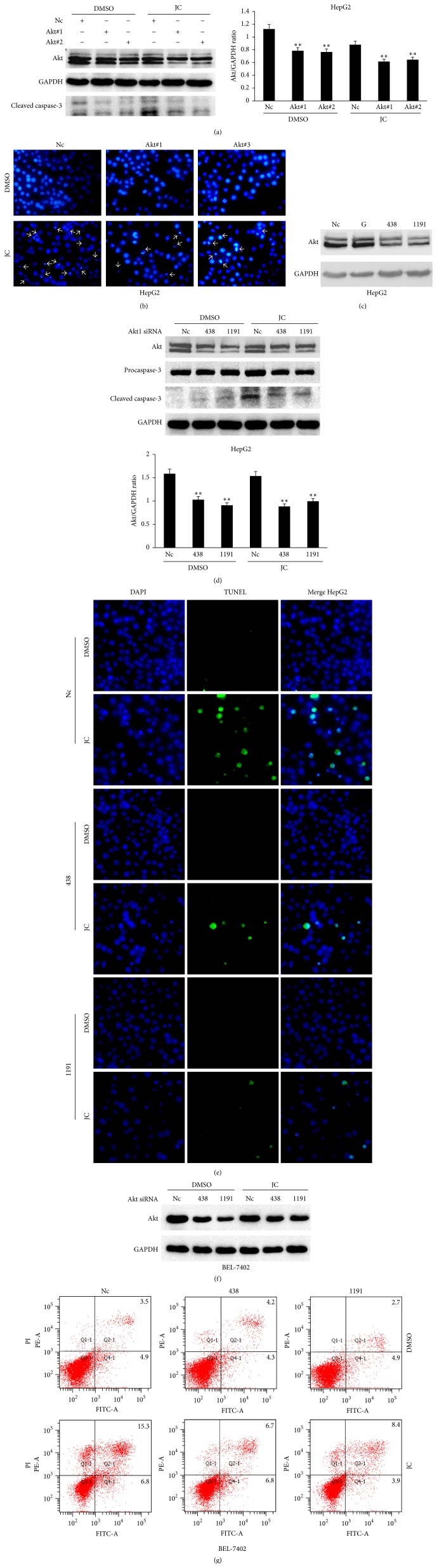
Akt deficiency abrogates HepG2 cell apoptosis induced by JC. (a) Effect of Akt deficiency on JC-induced apoptosis was determined by Western blot analysis. HepG2 cells were transfected with scrambled shRNA (Nc) or Akt shRNAs (Akt#1, Akt#2). Twelve hours after transfection, the cells were treated with either DMSO or 8 *μ*g/mL of JC for 36 hours. GAPDH was used as a loading control. The ImageJ software was used to quantify Akt levels. (b) Detection of apoptosis in JC-treated HepG2 cells by DAPI staining. HepG2 cells were transfected with scrambled shRNA (Nc) or Akt shRNAs (Akt#1, Akt#2). Twelve hours after transfection, the cells were treated with either DMSO or 8 *μ*g/mL of JC for 36 hours. The cells were fixed and stained with DAPI. Arrows are used to indicate apoptotic bodies in apoptotic HepG2 cells. (c) Effect of Akt siRNA on Akt expression was determined by Western blot analysis. HepG2 cells were transfected with scrambled siRNA (Nc) or Akt siRNAs (438, 1191) for 48 hours. G is GAPDH siRNA and is used as a positive control. GAPDH was used as a loading control. (d, e) Effect of Akt deficiency on JC-induced apoptosis. HepG2 cells were transfected with scrambled siRNA (Nc) or Akt siRNAs (438, 1191). Twelve hours after transfection, the cells were treated with either DMSO or 8 *μ*g/mL of JC for 36 hours. Then, cell apoptosis was detected by both Western blot analysis (d) and TUNEL staining (e). The ImageJ software was used to quantify Akt levels. (f, g) Effect of Akt deficiency on JC-induced apoptosis in BEL-7402 cells. The cells were transfected with scrambled siRNA (Nc) or Akt siRNAs (438, 1191). Twelve hours after transfection, the cells were treated with either DMSO or 8.7 *μ*g/mL of JC for 24 hours. Then, BEL-7402 cells were detected by both Western blot analysis (f) and flow cytometry analysis (g).

Akt is overactivated in a wide range of tumor types, and it triggers a cascade of responses, including cell growth, proliferation, survival, and motility, and drives tumor progression [25]. However, the aforementioned results have suggested that JC could induce apoptosis and increase the Akt phosphorylation level in HCC cells, which was contrary to the antiapoptotic effect of Akt. To verify whether Akt activation is required for JC-induced apoptosis, a shRNA interference approach was developed to selectively downregulate cellular Akt expression. Two shRNA plasmids against Akt were constructed and designated as Akt#1 and Akt#2, and Nc was a negative control. HepG2 cells were transfected with Akt#1 and Akt#2 and exposed to 8 *μ*g/mL of JC. As shown in Figure 3(a), compared with the control group, Akt deficiency obviously reduced the cleaved caspase-3 level and chromatin condensation induced by JC (Figures 3(a) and 3(b)).

To achieve a better interference effect than Akt shRNAs, HepG2 and BEL-7402 cells were infected with Akt siRNA, designated as 438 and 1191, and exposed to JC. This study suggested that Akt siRNAs resulted in an obvious reduction of Akt expression (Figures 3(c), 3(d), and 3(f)). Furthermore, the knockdown of Akt by siRNA greatly decreased the JC-induced cleaved caspase-3 level, as shown in Figure 3(d). Consistent with the preceding results, the TUNEL staining and flow cytometry analysis confirmed that Akt deficiencies significantly reduced the JC-induced apoptosis of HCC cells (Figures 3(e) and 3(g)). These results suggested that activating the Akt signaling pathway was required in JC-induced apoptosis in HCC cells.

### 3.4. Role of Akt Phosphorylation in JC-Induced Apoptosis

**Figure 4:**
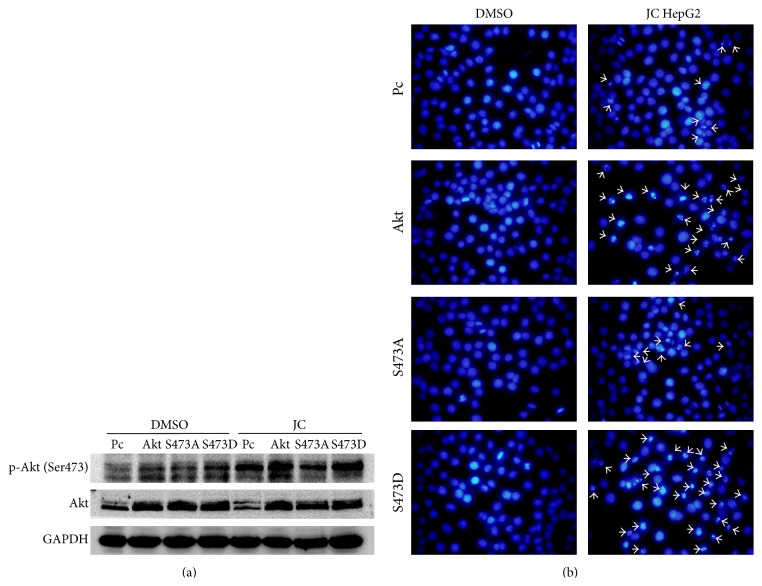
Akt activation promotes HepG2 cell apoptosis induced by JC. (a) Effects of wild-type and mutant Akt on JC-induced apoptosis were determined by Western blot analysis. HepG2 cells were transiently transfected with pcDNA3 vector control or pcDNA3-Akt. Twelve hours after transfection, the cells were treated with either DMSO or 8 *μ*g/mL of JC for 36 hours. In the Akt-WT group, the p-Akt (S473) blot is for both endogenous and exogenous Akt. In the other groups (PC, S473A, and S473D), the p-Akt (S473) blot is for the endogenous Akt. GAPDH was used as a loading control. (b) Effects of wild-type and mutant Akt on JC-induced apoptosis were determined by DAPI staining. Twelve hours after transfection, the cells were treated with either DMSO or 8 *μ*g/mL of JC for 36 hours. The cells were fixed and stained with DAPI. Arrows are used to indicate apoptotic bodies in apoptotic HepG2 cells.

Akt is a Ser/Thr kinase having a wide range of substrates. It is activated by recruitment to the plasma membrane, where it is phosphorylated at Thr308 and Ser473 [25]. It was shown that Akt was phosphorylated at Ser473 in JC-induced apoptosis. To further investigate the role of Akt phosphorylation in the apoptosis of HepG2 cells caused by JC, inactivated mutant of Akt (Akt-S473A), constitutively active Akt (Akt-S473D), and wild-type Akt (Akt-WT) were introduced in HepG2 cells to examine their effects on JC-induced apoptosis. Notably, the overexpression of Akt-S473A prevented apoptosis treated with JC in HepG2 cells, whereas the overexpression of Akt-WT and Akt-S473D caused higher levels of apoptosis, elucidating that JC regulated apoptosis at least partially through promoting the phosphorylation of Akt at Ser473 (Figures 4(a) and 4(b)).

### 3.5. Role of JC in Inducing Apoptosis, Activating Akt, and Inhibiting Foxo3a Transcriptional Activity

**Figure 5:**
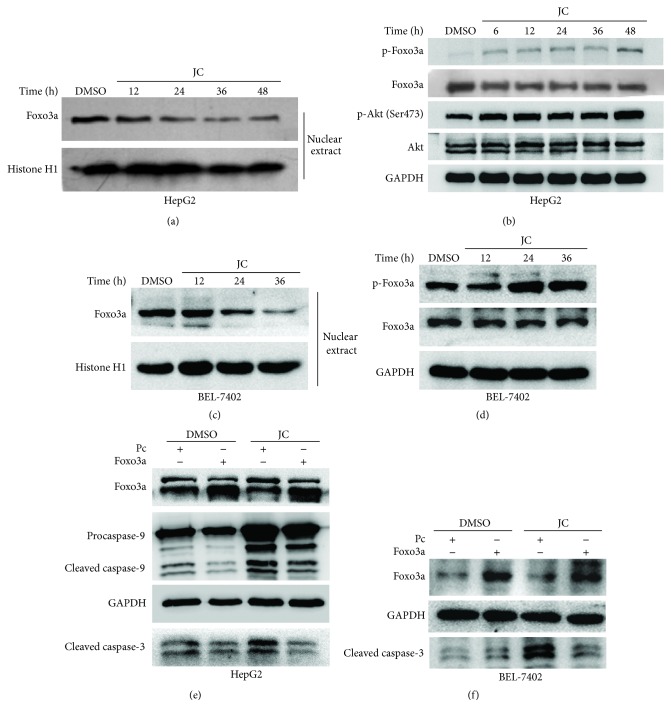
JC induces apoptosis by inhibiting the transcriptional activity of Foxo3a. (a, c) Effect of JC on Foxo3a levels in the nucleus was determined by Western blot analysis. HepG2 cells were treated with either DMSO or 8 *μ*g/mL of JC for the indicated times (a). BEL-7402 cells were treated with either DMSO or 8.7 *μ*g/mL of JC for the indicated times (c). Histone H1 was used as a loading control. (b, d) Effect of JC on the Akt/Foxo3a signaling pathway was determined by Western blot analysis. HepG2 cells were treated with either DMSO or 8 *μ*g/mL of JC for the indicated times (b). BEL-7402 cells were treated with either DMSO or 8.7 *μ*g/mL of JC for the indicated times (d). GAPDH was used as a loading control. (e, f) Overexpression of Foxo3a inhibits JC-induced apoptosis. HepG2 and BEL-7402 cells were transiently transfected with pcDNA3 vector control or pcDNA3-Foxo3a. Twelve hours after transfection, HepG2 cells were treated with either DMSO or 8 *μ*g/mL of JC for 36 hours (e). BEL-7402 cells were treated with either DMSO or 8.7 *μ*g/mL of JC for 24 hours (f). Then, cell apoptosis was detected by Western blot analysis. GAPDH was used as a loading control.

The Foxo family is a key downstream target of the PI3 K/Akt pathway and is directly phosphorylated by Akt [27]. The phosphorylation of Akt at Ser473 is required for its activation and inactivation of the Foxos [28]. The phosphorylation of Foxo factors by Akt triggers inactivation and rapid relocalization of Foxo proteins from the nucleus to the cytoplasm [29, 30]. The Foxo family participates in diverse processes including cell proliferation, apoptosis, stress resistance, differentiation, and metabolism [31]. The inactivation of Foxo transcription factors can induce apoptosis and regulate the cellular production of ROS [32]. Based on the aforementioned results and recent reports, whether Foxo3a translocated to the nucleus in JC-treated HepG2 and BEL-7402 cells was analyzed. As expected, it was found that JC treatment activated Akt, inhibited nuclear localization of Foxo3a, and increased phosphorylated Foxo3a levels (Figures 5(a)–5(d)).

To confirm the contribution of Foxo3a to the apoptosis induced by JC, a Foxo3a expression construct (pcDNA3-Foxo3a) was transfected into HepG2 and BEL-7402 cells. As shown in Figures 5(e) and 5(f), the overexpression of Foxo3a abrogated the apoptosis of HCC cells induced by JC, indicating that Foxo3a was necessary for the apoptosis induced by JC. All these data suggested that JC activated Akt signaling, inhibited the transcriptional activity of Foxo3a, and finally induced the apoptosis of HCC cells.

### 3.6. Effect of JC on ROS Levels

**Figure 6:**
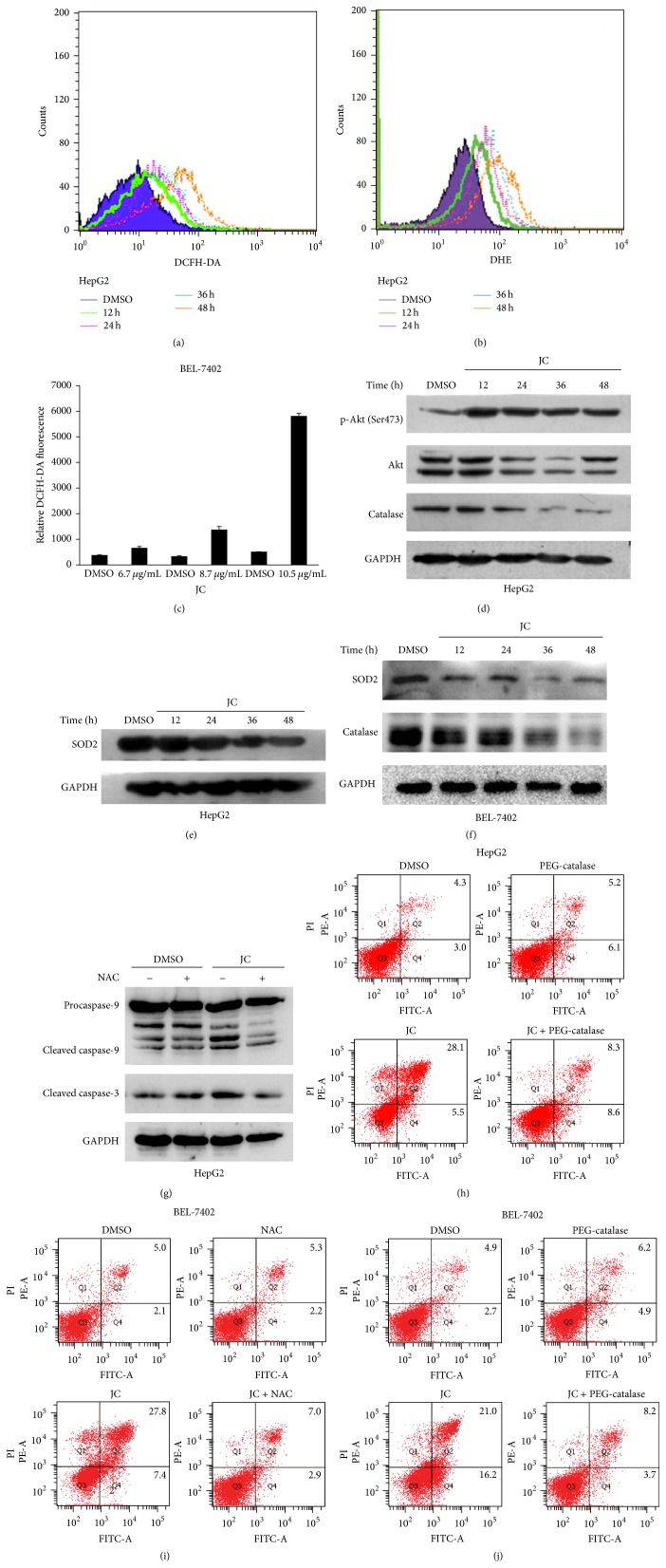
JC induces apoptosis by increasing intracellular ROS. (a, c) Effect of JC on the total ROS levels in HepG2 and BEL-7402 cells was examined by flow cytometry. HepG2 cells were treated with DMSO or 8 *μ*g/mL JC for 12, 24, 36, and 48 hours (a). BEL-7402 cells were treated with DMSO or 6.7, 8.7, and 10.5 *μ*g/mL of JC for 12 hours (c). (b) Effect of JC on superoxide anion levels in HepG2 cells was examined by flow cytometry. The levels of ROS in HepG2 cells were treated with either DMSO or 8 *μ*g/mL of JC for 12, 24, 36, and 48 hours and analyzed by flow cytometry. (d–f) Effects of JC on catalase and SOD2 expression and Akt activation were determined by Western blot analysis. HepG2 cells were treated with either DMSO or 8 *μ*g/mL of JC for the indicated times (d and e). BEL-7402 cells were treated with DMSO or 8.7 *μ*g/mL of JC for the indicated times (f). GAPDH was used as a loading control. (g–j) NAC and PEG-catalase abrogate JC-induced apoptosis. HepG2 cells were pretreated with NAC and PEG-catalase for 1 hour. HepG2 cells were treated with either DMSO or 8 *μ*g/mL of JC for 36 hours and detected by Western blot analysis (g) or flow cytometry analysis (h). GAPDH was used as a loading control. BEL-7402 cells were treated with either DMSO or 8.7 *μ*g/mL of JC for 24 hours and detected by flow cytometry analysis (i and j).

Foxos have effects on detoxification of ROS by upregulating the free radical scavenging enzymes, including SOD2 and catalase [33]. Results from Nogueira suggested that Akt had the ability to inhibit apoptosis induced by multiple apoptotic stimuli excluding ROS, and Akt activation sensitized cells to ROS-mediated apoptosis [13]. So, in this study, the effects of JC on the ROS levels in HepG2 and BEL-7402 cells were analyzed. After treating HepG2 cells with JC, the levels of intracellular ROS were evaluated by DCFH-DA or DHE staining separately. Compared with the control, treatment with JC significantly increased the level of ROS in a time-dependent manner (Figures 6(a) and 6(b)). Additionally, JC significantly increased ROS levels in BEL-7402 cells in a dose-dependent manner (Figure 6(c)).

Under normal conditions, low amounts of ROS levels were eliminated by scavenging enzymes such as SOD2 and catalase, which converted H_2_O_2_ to H_2_O and O_2_^−^ [34]. To verify whether JC increased ROS production by influencing SOD2 and catalase, their expressions were examined. As shown in Figures 6(d)–6(f), SOD2 and catalase were significantly decreased when cells were treated with JC for different times.

It was reported that treatment with antioxidant NAC and catalase was capable of restoring normal levels of intracellular ROS [33, 34]. To further determine whether the JC-induced apoptosis of HepG2 and BEL-7402 cells was mediated by increased ROS level, HCC cells were pretreated with NAC and PEG-catalase for 1 hour before incubating them with JC. This study showed that, compared to JC, NAC or catalase treatment could significantly reduce JC-induced apoptosis in HCC cells (Figures 6(g)–6(j)). These data suggested that treating HepG2 and BEL-7402 cells with JC resulted in high levels of ROS by inhibiting ROS scavenging, which severely damaged the cells and eventually led to the apoptosis of HCC cells.

## 4. Discussion

Natural products have long been a fertile source for cancer treatment drugs. At least 250,000 species of plants exist, out of which more than 1000 plants were found to possess significant anticancer properties [35]. Many molecules obtained from the nature have shown anticancer activity and have become anticancer agents in clinical practice, such as paclitaxel [36], podophyllotoxin [37], camptothecin [38], and so on. This study has shown that JC, an anthraquinone compound extracted from* J. mandshurica* Maxim, could induce the apoptosis of cancer cells. In this study, a comparison of the three GO terms related to cell death showed that the shared 81 probe sets were identified in the three terms (Figure 1(d)). It can be inferred that these genes may be related to the apoptosis of HepG2 cells.

RTKs are the main type of enzyme-linked receptors that played an important role in the development and progression of cancer [39]. RTKs can activate MAPK, NF-*κ*B, and PI3 K/Akt signaling pathways. In cancer cells, Akt activation promotes cell proliferation, regulates cellular energy metabolism, and provides protection from apoptosis, which could partly explain that it is frequently activated in human cancers [12]. Here, whether JC induced apoptosis by inhibiting these signaling pathways was investigated. Surprisingly, the results from microarrays showed that enzyme-linked receptor protein signaling pathways were activated and the genes positively regulating Akt signaling were upregulated, after treatment with JC. The Western blot analysis further confirmed that Akt was activated after treatment with JC, which induced the apoptosis of HepG2 and BEL-7402 cells (Figure 2). This study also demonstrated that Akt deficiency obviously inhibited apoptosis while the overexpression of a dominant-active mutant of Akt accelerated apoptosis induced by JC (Figures 3 and 4), suggesting that HepG2 cells that were transfected with Akt shRNA and siRNA were less sensitive than WT cells to JC-induced apoptosis. All these results suggested that JC-induced apoptosis was mediated by Akt activation, and this result was different from that of a previous study [12].

Akt normally acted as a proliferative signal, but the role of Akt is also a double-edged sword. Hyperactivated Akt also attenuates G2 arrest in Rat1a cells following DNA damage and induces premature senescence and sensitizes cells to ROS-mediated apoptosis [13, 40]. Aberrant loss or gain of Akt activation underlies the pathophysiological properties of a variety of complex diseases, including cancer [27, 41]. So the hypothesis that Akt activation was required for JC-induced apoptosis was investigated.

Akt is recruited to the plasma membrane by phosphatidylinositol-3,4,5-triphosphate and phosphorylated by 3-phosphoinositide-dependent protein kinases 1 and 2 at Thr308 and Ser473, respectively, which causes the full activation of Akt [25]. Activated Akt phosphorylates a wide range of direct intracellular targets containing a minimal Akt recognition motif, including Bad, Tsc2, Gsk3, and Foxos, which contribute to the diverse cellular roles of Akt, including cell survival, growth, proliferation, metabolism, and migration [27, 30]. Akt phosphorylation at Ser473 is required for the inactivation of the Foxos [28]. After being activated, Akt directly phosphorylates Foxos, and this phosphorylation excludes Foxos from the nucleus, thereby inhibiting their transcriptional activity [42]. It was found that the transcription activity of Foxo3a was inhibited after treatment with JC (Figures 5(a)–5(d)). Additionally, the apoptosis of HCC cells induced by JC was abrogated by the overexpression of Foxo3a (Figures 5(e) and 5(f)), suggesting that Foxo3a was a key factor in regulating JC-induced apoptosis and Akt activation.

ROS are generally small, short-lived, and highly reactive molecules, formed by incomplete one-electron reduction of oxygen [14]. The damage induced by the accumulation of ROS is considered a major determinant of life span at both the organismal and cellular levels. ROS can damage proteins, nucleic acids, and intracellular membranes, which lead to oxidative stress and impairment of cellular functions [14]. Excessive ROS causes the release of Cytochrome* c* from mitochondria to the cytosol and triggers caspase-9 activation and apoptosis [15]. In this study, the levels of intracellular ROS were evaluated after cells were treated with JC (Figures 6(a) and 6(c)). The increase of apoptosis induced by JC was reversed by the antioxidant NAC and PEG-catalase (Figures 6(g)–6(j)). Therefore, these results indicate that JC-induced mitochondrial apoptosis is mediated by ROS.

ROS scavengers SOD2 and catalase are known to be Foxo target gene. Under normal conditions, ROS are reduced by nonenzymatic and enzymatic antioxidizing agents, such as glutathione, thioredoxin, SOD, catalase, and peroxidases [14, 34]. In this study, both SOD2 and catalase were significantly decreased, while ROS levels were increased, when HCC cells were treated with JC (Figure 6). These results suggest that SOD2 and catalase are related to the increased ROS levels induced by JC. Akt could also increase ROS levels by increasing oxygen consumption. Most of ROS are products of mitochondrial respiration and generated at Complexes I and III of the respiratory chain [14, 43]. Akt can increase cellular ATP production by accelerating both glycolytic and oxidative metabolism [44], which contributes to an increase of ROS levels.

Given that activating the PI3 K/Akt pathway is frequently implicated in human cancer, many intracellular components of the PI3 K/Akt pathway have been targeted as anticancer drug discovery [45]. However, existing drugs against various components of the PI3 K/Akt pathway possibly exhibit undesired physiological consequences such as diabetes. Compared with normal cells, cancer cells normally contain higher levels of ROS, which can stimulate cell proliferation and induce genetic instability [46]. It was reported that abnormal increases in ROS can be exploited to selectively kill cancer cells [47]. Thus, using hyperactivated Akt and high levels of ROS as targets is a strategy to selectively kill cancer cells. It was demonstrated that JC can selectively eradicate HepG2 and BEL-7402 cells with hyperactivated Akt by inducing excessive ROS, suggesting that JC is a potentially effective anticancer drug.

It was reported that the activation of Akt is frequently implicated in resistance to anticancer drugs [48]. Moreover, this study proved that JC can selectively kill HCC cells with hyperactivated Akt. So the combination of JC and anticancer drugs, such as PEITC and rapamycin, could be an effective strategy to selectively eradicate tumors that display hyperactive Akt and resistance to anticancer drugs.

**Figure 7:**
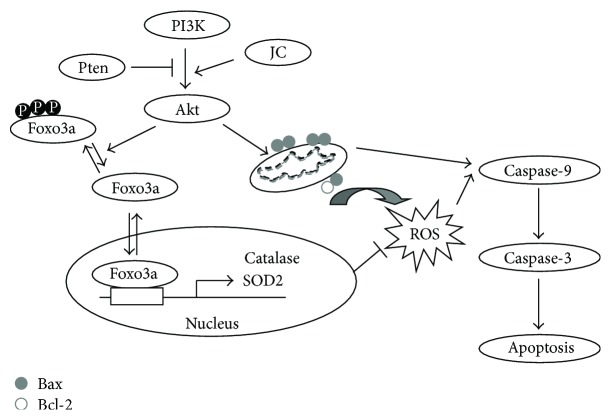
A proposed model to illustrate mechanisms by which JC induced the apoptosis of HCC cells. Akt hyperactivation caused by JC-inhibited Foxo transcriptional activity, which resulted in highly increased ROS levels via impairment of ROS scavenging. The upregulation of ROS, in turn, promoted apoptosis of HepG2 and BEL-7402 cells.

Overall, these findings suggest a model (Figure 7) in which JC increases Akt Ser473 and Foxos phosphorylation. Foxos were excluded from the nucleus, thereby inhibiting the expression of their target genes SOD2 and catalase, resulting in the intracellular ROS accumulation, and eventually leading to cell apoptosis.
